# Effect of student-directed solicitation of evaluation forms on the timeliness of completion by preceptors in the United States

**DOI:** 10.3352/jeehp.2019.16.32

**Published:** 2019-10-16

**Authors:** Conrad Krawiec, Vonn Walter, Abigail Kate Myers

**Affiliations:** 1 Pediatric Critical Care Medicine, Department of Pediatrics, Penn State Children’s Hospital, Hershey, PA, USA; 2 Department of Public Health Sciences, Pennsylvania State College of Medicine, Hershey, PA, USA; 3 General Pediatrics, Department of Pediatrics, Penn State Children’s Hospital, Hershey, PA, USA; Hallym University, Korea

**Keywords:** Educational measurement, Educational technology, Medical education, Reminder systems, United States

## Abstract

**Purpose:**

Summative evaluation forms assessing a student’s clinical performance are often completed by a faculty preceptor at the end of a clinical training experience. At our institution, despite the use of an electronic system, timeliness of completion has been suboptimal, potentially limiting our ability to monitor students’ progress. The aim of the present study was to determine whether a student-directed approach to summative evaluation form collection at the end of a pediatrics clerkship would enhance timeliness of completion for third-year medical students.

**Methods:**

This was a pre- and post-intervention educational quality improvement project focused on 156 (82 pre-intervention, 74 post-intervention) third-year medical students at Penn State College of Medicine completing their 4-week pediatric clerkship. Utilizing REDCap (Research Electronic Data Capture) informatics support, student-directed evaluation form solicitation was encouraged. The Wilcoxon rank-sum test was applied to compare the pre-intervention (May 1, 2017 to March 2, 2018) and post-intervention (April 2, 2018 to December 21, 2018) percentages of forms completed before the rotation midpoint.

**Results:**

In total, 740 evaluation forms were submitted during the pre-intervention phase and 517 during the post-intervention phase. The percentage of forms completed before the rotation midpoint increased after implementing student-directed solicitation (9.6% vs. 39.7%, P<0.05).

**Conclusion:**

Our clerkship relies on subjective summative evaluations to track students’ progress, deploy improvement strategies, and determine criteria for advancement; however, our preceptors struggled with timely submission. Allowing students to direct the solicitation of evaluation forms enhanced the timeliness of completion and should be considered in clerkships facing similar challenges.

## Introduction

### Background

Medical schools are required to have fair and timely summative assessments of students [1]. Summative assessments are comprehensive and high-stakes, as they determines whether a student is ready to advance to the next level of his or her medical training [2]. One component of the summative assessment is the preceptor, who works closely with students, teaches core medical knowledge and concepts, and provides important formative feedback [3]. This active engagement with students throughout a clinical experience helps determine whether a student can provide high-quality patient care [4]. Thus, individual subjective summative evaluations are often solicited directly from the preceptor, who then assesses how well a student performed based on the competencies of a physician [5].

In core clerkships, while controversial, the collection of multiple subjective summative evaluation forms from different preceptors is often an element considered to determine advancement [6]. Lack of timely completion may limit a clerkship’s ability to assess a student’s performance [1]. While preceptors have a duty to provide an accurate summative assessment, doing so may be considered a lower priority, especially in busy clinical work environments [7,8].

### Objectives

We proposed that students undertake the responsibility to assist their preceptors in ensuring the timely completion of subjective summative evaluations of their clinical performance. The aims of this quality improvement study were to describe our experiences in developing a student-centered method to solicit subjective summative evaluation forms and to demonstrate how this method was used to enhance the timeliness of form completion.

## Methods

### Ethics statement

This study was reviewed by the Institutional Review Board at the Penn State College of Medicine in Hershey, PA, USA (STUDY00010320) and was determined to be non-clinical research because it was a retrospective review of evaluation forms submitted by preceptors.

### Study design

**Fig. 1.**
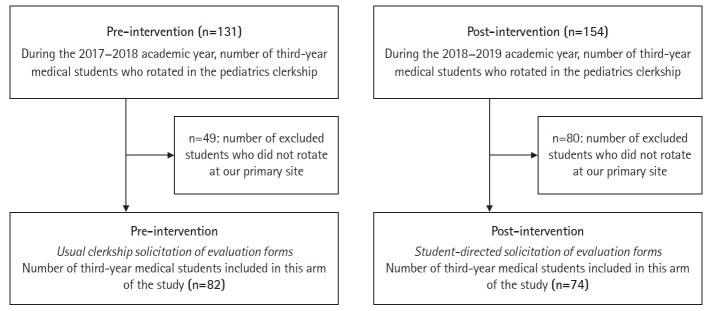
Flow diagram of participants in this study on student-directed solicitation of evaluation forms.

This was a pre- and post-intervention quality improvement study focused on improving the timeliness of summative evaluation form completion by preceptors (Fig. 1).

### Setting

A retrospective review of submitted subjective summative evaluation forms was completed during the 2017–2018 academic year. These findings were then compared to data collected after implementation of the intervention during the 2018–2019 academic year, on May 7, 2019.

### Materials

The study materials were evaluation form data from 156 third-year medical students at Penn State College of Medicine who were assigned to complete their required 4-week pediatric clerkship at our primary clinical site (outpatient and inpatient) during the 2017–2018 academic year (82 students; pre-intervention) and the 2018–2019 academic year (74 students; post-intervention).

### Usual summative assessment process

The pediatric clerkship utilized the Online Access to Student Information and Scheduling (OASIS; Schilling Consulting, Madison, WI, USA) electronic system to submit and store subjective summative evaluations. These evaluation forms are routinely assigned to preceptors (attending physicians and residents) at the end of a clinical training experience. The assignments were determined by the student providing the preceptor’s name to the clerkship coordinator or by the clerkship coordinator identifying the preceptor via the schedule. Once an assignment was made, an automated electronic reminder occurred to encourage the preceptor to complete the evaluation form.

### REDCap subjective summative evaluation form solicitation intervention

The primary intervention was directing the student to invite the preceptor to complete a summative evaluation form at the end of the clinical experience. To increase the likelihood of completion, the student was encouraged to establish learning goals and expectations with their preceptor prior to the start of the clinical experience and to solicit formative feedback throughout the clinical experience. At the time of summative evaluation form solicitation, the student accessed a website link on a mobile electronic device to open the Research Electronic Data Capture (REDCap)–based subjective summative evaluation tool. REDCap is a secure web-based application that has the capability to collect summative assessment data from a preceptor [9]. After the form was accessed, the device was provided to the preceptor to be completed privately.

### Development of the REDCAP electronic subjective summative evaluation tool

The REDCAP electronic subjective summative evaluation tool mimicked the form used in OASIS (Supplement 1). This web-based tool was accessible via a public link and provided the preceptor 3 different options during form completion: first, to defer if the amount of time to allow form completion was inadequate, thus triggering our clerkship coordinator to assign a form to the preceptor via the OASIS platform for completion at a later date; second, form refusal if the preceptor felt that he or she was unable to provide a summative evaluation due to having spent inadequate time with the student; and third, form completion, in which the preceptor would complete the form and once electronically collected, our clerkship coordinator would enter the results into OASIS. Once a week, all preceptors who completed forms in this manner, would be notified to review the entered OASIS evaluation forms and attest to their accuracy. Each preceptor was required to provide text describing the student’s areas of strength and opportunities for improvement. All preceptors and students were notified that the subjective summative evaluation forms would be considered as part of a summative assessment of the student’s clerkship performance.

### Intervention, implementation, and alterations

After developing this intervention and the REDCAP electronic subjective summative evaluation tool, it was implemented on April 2, 2018. This process was introduced to students via e-mail and then discussed on the first day of the pediatric clerkship during orientation. Preceptors were introduced to this new process via e-mail. A run-in period was applied during the first month to determine whether this process and study were feasible. The results from this period were included in the study. A minimum amount of forms (1 from a resident and 1 from an attending physician) were asked to be completed starting on April 30, 2018 due to students’ requests for clearer guidance.

### Data collection

In the pre-intervention phase, using the OASIS system, the subjective summative evaluation forms for students who completed the pediatric clerkship were obtained from May 1, 2017 to March 2, 2018. For the post-intervention phase, the subjective summative evaluation forms for students who completed the pediatric clerkship were obtained from April 2, 2018 to December 21, 2018. We quantified the total number of students for each 4-week rotation, the forms submitted, evaluator type (attending or resident), time of submission, and the number of forms that were completed prior to the midpoint feedback session of the pediatric clerkship, before the end of the rotation, and after completion of the rotation. Evaluation forms were included in this study only if the REDCap tool was used. If duplicate forms were completed by the same preceptor, both were only counted if the duplicate was determined to be entered as a follow-up assessment of a student’s performance. If a duplicated form was entered in error, it was only counted once. These determinations were made after discussion of the nature of the subjective summative evaluation form with the preceptor via e-mail. The study data were collected and managed using REDCap electronic data capture tools hosted at Penn State Health Milton S. Hershey Medical Center and Penn State College of Medicine [9]. It was continuously utilized during the course of the study to track student compliance with the system, to determine whether there were any issues during solicitation, and to identify errors. In addition, because the REDCap tool could be publicly accessed (i.e., the evaluation form was not directly assigned to a particular preceptor), there was a possibility that students might self-enter their own evaluations in an attempt to falsify records of their performance. Because of this, we continuously reviewed several variables that could indicate a falsified entry (i.e., inconsistent evaluation scores, timing of evaluation entry, evaluations entered after the final grade was submitted, and preceptors not recalling completion of the evaluation form).

### Outcome measurement

The following outcome measures were computed: the total number of subjective summative evaluation forms completed and the percentage of forms completed before the midpoint of the rotation (14 days after the start of the clerkship). Process measures that were tracked included the percentage of forms that were ultimately submitted as a direct consequence of utilizing the REDCAP electronic tool, the percentage of forms completed before the end of the rotation, and the percentage of forms submitted after completion of the rotation. The percentage of attendings and residents completing the subjective evaluation forms and the occurrence of evaluations with scores equal to or less than 2, utilized by our institution as an indication that a student may be struggling, were analyzed as balancing measures.

### Statistical methods

The Wilcoxon rank-sum test was applied to compare the pre-intervention percentages and the post-intervention percentages. R version 3.5.0 (R Development Core Team, The R Foundation for Statistical Computing, Vienna, Austria) was used to perform all data analyses on May 7, 2019 [10].

## Results

### Utilization of the REDCAP electronic tool

The REDCAP tool was utilized 593 times, of which 553 (93.3%) forms were completed correctly. Twenty-four forms were unintentional duplications and 16 forms were incomplete. Of the correctly completed forms, 378 (68.4%) subjective evaluation forms were completed at the time of solicitation, and 2 (0.4%) were deferred due to a perceived lack of time with the student. One additional form was noted to be deferred due to perceived lack of time with the student, but then was ultimately completed later outside of our methodological framework. This form was not included in our final analysis. Overall, 169 solicitations (30.6%) resulted in a request to receive an OASIS subjective evaluation form to be completed at a later date. Of these, 135 (24.4%) were ultimately completed via OASIS. Three (0.5%) solicitations were deferred due to an unspecified reason, but another solicitation was requested within the rotation, at which point it was completed.

### Subjective summative evaluation form submission

In total, 740 forms were submitted during the pre-intervention phase, and 558 valid forms were submitted during the post-intervention phase (Dataset 1). Eight forms were unintentionally entered twice into the OASIS system and were thus removed from the final analysis. Thirty-three forms were excluded because they were not obtained using our new methodology. In sum, 517 (92.7%) of the forms in the post-intervention period were submitted utilizing the REDCap tool and were included in the final analysis.

### Subjective summative evaluation form submission prior to the clerkship midpoint feedback session

**Fig. 2.**
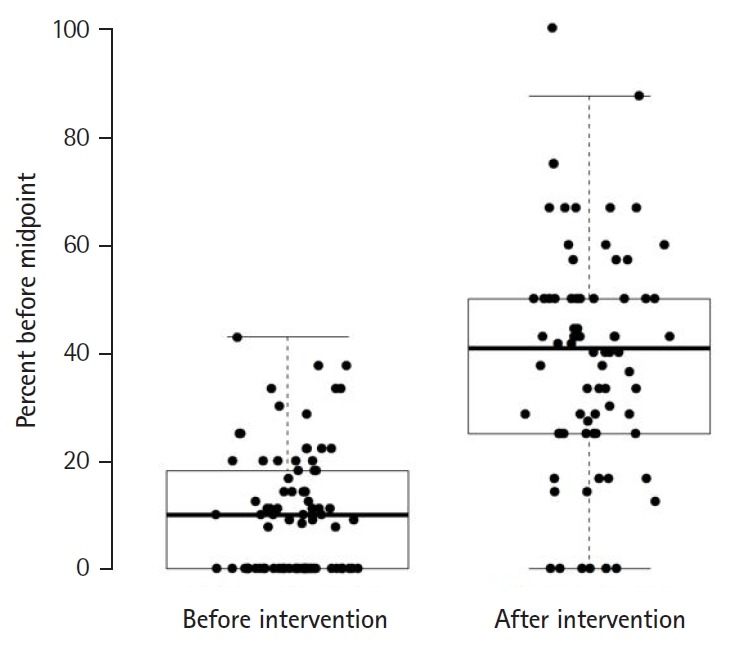
Comparison of the percentage of forms submitted before the rotation midpoint before and after the intervention.

Seventy-one (9.6%) forms were submitted prior to the clerkship midpoint feedback session in the pre-intervention period and 205 (39.7%) forms were submitted prior to the midpoint in the post-intervention period (P<0.05) (Table 1, Fig. 2).

### Subjective summative evaluation forms submitted after the rotation midpoint

The percentage of forms submitted after the rotation midpoint, but before the end of the rotation, were similar in the pre-intervention (188, 25.4%) and post-intervention groups (137, 26.5%). The percentage of forms submitted after the end of the rotation was higher in the pre-intervention group (481, 65%) than in the post-intervention group (175, 33.8%) (Table 1).

### Preceptor category

In the pre-intervention period, a total of 463 (63.6%) residents and 277 (37.4%) attendings completed forms, while 277 (53.6%) residents and 240 (46.4%) attendings completed forms in the post-intervention period (Table 1).

### Occurrence of concerning subjective summative evaluation form submissions

In the pre-intervention period, 5 students received concerning subjective summative evaluations. In the post-intervention period, 8 students received concerning subjective summative evaluations (Table 1).

## Discussion

### Key results

We hypothesized that by providing them with directed guidance for their clerkships and extending more autonomy, we would encourage students to identify preceptors, solicit subjective summative evaluations of their performance, and increase the timeliness of form submission. This quality improvement project successfully introduced a student-driven way to increase the amount of subjective summative evaluations before a midpoint clerkship review session at an academic center (Fig. 2). By empowering students to collect data on their own performance, this intervention provided the opportunity to track students’ progress during the clerkship and determine their likelihood for progression.

### Interpretations

While the weight that clerkships apply to a student’s overall performance varies, a subjective summative evaluation is still a common element reviewed when determining whether a student meets the criteria for progression [6]. Such evaluations allow the preceptor to directly communicate to the clerkship director their assessment of a student’s performance and ability to practice patient care [4]. Subjective summative evaluations at the end of a clinical experience by faculty with whom students have closely worked can potentially drive learning and allow students to identify opportunities for improvement as they progress to another clinical environment [11]. If a student is struggling, it may be better to identify this early on, as the student may have continuing issues as they rotate through other clinical experiences or other clerkships.

However, summative clinical assessments have limitations. They are subjective, can be unreliable if there is an inadequate amount of data available, and when they are utilized as part of a grade, the student may not show any growth in learning [4]. Alternative methods of determining a student’s performance should continue to be researched and evaluated, but until this occurs, clerkships should continue to strive to maximize the reliability of subjective summative evaluations. This is accomplished by encouraging preceptors to complete these evaluation forms in a timely manner and to keep track of the amount of forms completed to ensure an adequate quantity. By collecting numerous data points during a student’s progress through the clerkship, with sufficient time to review the submitted forms, our clerkship was able to increase the likelihood of providing a meaningful appraisal of a student’s performance [4].

Our study highlighted the potential weaknesses of using an electronic system to solicit subjective summative evaluation forms. Creation of these systems was a necessary improvement from paper-based forms, as it allowed an easier way to review a trainees’ performance. However, this electronic enhancement required less human interaction and depended on automation to ensure form completion. While convenient, it may have also made it easier for our faculty to defer and ignore a computer prompt, especially if requested to complete a subjective summative evaluation form after the student leaves a clinical service. Any future innovations in methods of assessing students in the clinical work environment should take into account the weaknesses of utilizing an electronic system.

Although the method formalized herein can potentially increase the number of subjective summative evaluation forms that a student receives in a timely manner, it is unclear whether this correlates with students’ ability to provide high-quality clinical care. Dudas et al. demonstrated that in a 9-week pediatric clerkship at 1 academic center, assessments by attendings and residents correlated with students’ clinical knowledge on the National Board of Medical Examiners exam [12]. Recently, Dubosh et al. [13] demonstrated that faculty summative evaluation forms correlated poorly with students’ written examination performance during a 4-week emergency medicine clerkship. While these differences could have been due to the time spent with the preceptors during the clerkship, with more time increasing the likelihood that more meaningful information could be obtained on a student’s clinical performance [13], the methodology of form distribution may have also been a factor.

### Limitation

This was a single-institution study. Because this tool was student-directed, it is unknown whether the guidance provided was followed, including whether formative feedback was prioritized or completed at the time of solicitation. During this study, informal feedback was obtained from the students and the faculty to optimize the tool, but none was collected formally to determine whether this tool was favored over other methods. These evaluations are considered as a part of the grade system, and students may therefore have chosen preceptors who they perceived had a high likelihood of providing a favorable evaluation. Overly focusing on their grade may cause some students to fixate on task completion, rather than identifying improvement opportunities. Finally, there was a risk of students self-entering their own evaluations, potentially falsifying their performance. Our clerkship relied on the honor system and the belief that medical students who are training to be physicians would not dishonor themselves. However, because this was not a guarantee, our clerkship conservatively developed strategies to reduce this risk. To our knowledge, we have not identified any falsified data.

### Conclusion

To be able to monitor students’ progress and determine criteria for advancement, clerkships may require subjective summative evaluations from preceptors who closely work with students. Lack of timely completion may limit a clerkship’s ability to track students’ progress and to deploy improvement strategies. Allowing students to direct the solicitation of subjective summative evaluations may facilitate this process.

## Figures and Tables

**Table 1.** Overview of the timing of evaluation form submissions by preceptors for medical students at the Penn State College of Medicine during academic years 2017–2018 (pre-intervention) and 2018–2019 (post-intervention)

Variable	Pre-intervention	Post-intervention
Total no. of students	82	74
Total no. of included forms completed	740	517
Forms submitted before rotation midpoint	71 (9.6)	205 (39.7)^*^
Forms submitted before rotation end	188 (25.4)	137 (26.5)
Forms submitted after rotation completion	481 (65.0)	175 (33.8)
Resident submissions	463 (63.6)	277 (53.6)
Attending submissions	277 (37.4)	240 (46.4)
Occurrence of red flag submissions	-	2

Values are presented as number (%).

* P<0.05.

## Footnotes

**Data availability** Data files are available from https://doi.org/10.7910/DVN/QCGSND Dataset 1. Data of completion of evaluation forms by preceptors for medical students’ pediatrics clerkship at Penn State College of Medicine, the United States.

## Supplementary materials

Data files are available from https://doi.org/10.7910/DVN/QCGSND

Supplement 1. Screenshot of the REDCap Electronic Summative Subjective Evaluation Tool

Supplement 2. Audio recording of the abstract.
