# Supplementary material for: Effect of student-directed solicitation of evaluation forms on the timeliness of completion by preceptors in the United States
Source: J Educ Eval Health Prof. 2019 Oct 16;16:32. doi: 10.3352/jeehp.2019.16.32 (PMC6819957; doi:10.3352/jeehp.2019.16.32)
Supplement: Supplementary file 1 — Supplement 1. Screenshot of the REDCap Electronic Summative Subjective Evaluation Tool [file jeehp-16-32-suppl.pdf]

Supplement 1. Screenshot of the REDCap Electronic Summative Subjective Evaluation Tool

Please complete the evaluation below.

Thank you!

Please enter student's name.	__________________________
Please enter preceptor name.	__________________________
Do you want to complete this evaluation at this time?	○ Yes<br>○ No
Please provide a reason why you do not want to complete this evaluation at this time.	☐ I do not feel that I had enough time with this student to complete an evaluation<br>☐ I do not feel that I have time right now, but please send me the Oasis Clinical Assessment evaluation form later this week<br>☐ Other

Keeping in mind this clerkship student's level of training, please indicate the student's demonstration of the following behaviors below.

KNOWLEDGE FOR PRACTICE:

Based on this student's level of training, indicate this student's demonstration of his/her use of appropriate medical science concepts (basic and clinical sciences) to justify his/her diagnostic decisions and treatment plans.

- ○ 1= Struggles to recall basic knowledge of biomedical concepts even with prompting
- ○ 2= Recalls basic knowledge of biomedical concepts with significant prompting. Recognizes normal and abnormal states. Unable to connect biomedical sciences to the patient case.
- ○ 3= Recalls basic knowledge of biomedical concepts when prompted. Superficially connects biomedical sciences to diagnostic and therapeutic decision making.
- ○ 4= Explains biomedical concepts with little prompting. Is able to apply this knowledge to diagnostic and therapeutic decision making.
- ○ 5= Independently explains biomedical science concepts. Routinely applies knowledge to diagnostic and therapeutic decision making.
- ○ N/A= I did not have sufficient interaction with this student to assess this behavior

Please provide comments:	__________________________

HISTORY AND PHYSICAL EXAM:

Based on this student's level of training, indicate this student's demonstration of his/her ability to gather essential and accurate information about patients and their conditions through history-taking and physical examination.

- ○ 1= Significant gaps in history and exam skills. Relies solely on external data sources (medical record, nursing notes, etc.)
- ○ 2= Requires substantial guidance to obtain relevant details. Incorrectly performs exam maneuvers despite feedback.
- ○ 3= Able to conduct a basic history and exam. Several omissions of relevant HPI/PMH/PSH/FH/SH.
- ○ 4= Accurate, reliable and efficient but may need guidance in selecting a focused or complete strategy. Includes most of the relevant HPI/PMH/PSH/FH/SH and ROS.
- ○ 5= Accurate, reliable and efficient. Appropriately selects focused or complete history and exam strategy. Includes all relevant HPI/PMH/PSH/FH/SH and ROS.
- ○ N/A= I did not have sufficient interaction with this student to assess this behavior
